# Chronic TNF exposure induces glucocorticoid‐like immunosuppression in the alveolar macrophages of aged mice that enhances their susceptibility to pneumonia

**DOI:** 10.1111/acel.14133

**Published:** 2024-03-08

**Authors:** Katherine L. Kruckow, Elizabeth Murray, Elnur Shayhidin, Alexander F. Rosenberg, Dawn M. E. Bowdish, Carlos J. Orihuela

**Affiliations:** ^1^ Department of Microbiology University of Alabama at Birmingham Birmingham Alabama USA; ^2^ Firestone Institute for Respiratory Health St. Joseph's Healthcare Hamilton Hamilton Ontario Canada; ^3^ The M.G. DeGroote Institute for Infectious Disease Research McMaster University Hamilton Ontario Canada; ^4^ Informatics Institute University of Alabama at Birmingham Birmingham Alabama USA

**Keywords:** alveolar macrophages, Dusp1, glucocorticoids, inflamm‐aging, MAPK, pneumonia, Ptprs, *Streptococcus pneumoniae*, tumor necrosis factor

## Abstract

Chronic low‐grade inflammation, particularly elevated tumor necrosis factor (TNF) levels, occurs due to advanced age and is associated with greater susceptibility to infection. One reason for this is age‐dependent macrophage dysfunction (ADMD). Herein, we use the adoptive transfer of alveolar macrophages (AM) from aged mice into the airway of young mice to show that inherent age‐related defects in AM were sufficient to increase the susceptibility to *Streptococcus pneumoniae*, a Gram‐positive bacterium and the leading cause of community‐acquired pneumonia. MAPK phosphorylation arrays using AM lysates from young and aged wild‐type (WT) and TNF knockout (KO) mice revealed multilevel TNF‐mediated suppression of kinase activity in aged mice. RNAseq analyses of AM validated the suppression of MAPK signaling as a consequence of TNF during aging. Two regulatory phosphatases that suppress MAPK signaling, *Dusp1* and *Ptprs*, were confirmed to be upregulated with age and as a result of TNF exposure both ex vivo and in vitro. Dusp1 is known to be responsible for glucocorticoid‐mediated immune suppression, and dexamethasone treatment increased *Dusp1* and *Ptprs* expression in cells and recapitulated the ADMD phenotype. In young mice, treatment with dexamethasone increased the levels of *Dusp1* and *Ptprs* and their susceptibility to infection. TNF‐neutralizing antibody reduced *Dusp1* and *Ptprs* levels in AM from aged mice and reduced pneumonia severity following bacterial challenge. We conclude that chronic exposure to TNF increases the expression of the glucocorticoid‐associated MAPK signaling suppressors, *Dusp1* and *Ptprs,* which inhibits AM activation and increases susceptibility to bacterial pneumonia in older adults.

## Graphical abstract

Elevated TNF levels because of advanced age increase the expression of *Dusp1* and *Ptprs*, which encode negative regulators of MAPK signaling. This inhibits the ability of alveolar macrophages to effectively respond to bacterial infection. This TNF‐mediated suppression phenocopies glucocorticoid immunosuppression, which has been shown to be mediated by *Dusp1*.

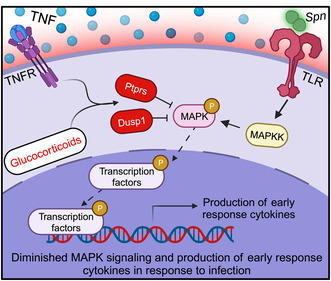

**Abbreviations**
ADMD: age‐dependent macrophage dysfunction
AM: alveolar macrophages
BALF: bronchoalveolar lavage fluid
DEGs: Differentially‐expressed genes
Dusp1: Dual Specificity Phosphatase 1
EkSpn: ethanol‐killed Spn
IPA: Ingenuity Pathway Analysis
IL: Interleukin
KO: Knockout
MIP‐2, also known as CXCL2: macrophage inflammatory protein 2
MPO: myeloperoxidase
PBS: phosphate‐buffered saline
PMN: polymorphonuclear cell
Ptprs: Protein Tyrosine Phosphatase Receptor Type S
ROS: reactive oxygen species
Spn, the pneumococcus: Streptococcus pneumoniae
TNF: Tumor necrosis factor

## 1 INTRODUCTION

Aging is associated with the remodeling of the immune system with a progressive tendency toward a more pro‐inflammatory phenotype alongside an inability to fine‐tune the response and resolve systemic inflammation, a phenomenon referred to as “inflamm‐aging” (Franceschi et al., 2000). Often, aging results in a dysregulated cytokine network with persistently elevated levels of many chemokines/cytokines and inflammatory mediators, including but not limited to tumor necrosis factor (TNF), interleukin‐6 (IL‐6), IL‐1 receptor antagonist (IL‐1ra), reactive oxygen species (ROS), and acute phase proteins such as C‐reactive protein (CRP; Ferrucci et al., 2005; Marcos‐Pérez et al., 2020; O'Mahony et al., 1998; Varadhan et al., 2014). This chronic low‐grade inflammation is damaging and, in older adults, has been associated with increased morbidity, the onset of frailty, and mortality (Bruunsgaard et al., 1999, 2003; Cohen et al., 1997; de Gonzalo‐Calvo et al., 2012; Fried et al., 2001; Harris et al., 1999; Mooradian et al., 1991; Varadhan et al., 2014; Volpato et al., 2001). Inflamm‐aging drives the pathophysiological mechanisms underlying many age‐related conditions, including progressive neurodegenerative diseases, atherosclerosis, diabetes mellitus, cardiovascular disease, and cancer (Bae et al., 2017; Beutler et al., 1985; Bruunsgaard et al., 1999, 2003; Fillit et al., 1991; Firestein & McInnes, 2017; McInnes & Schett, 2007; Van Deventer, 1997). Inflamm‐aging also enhances susceptibility to infectious diseases, including pneumonia, which is the leading cause of infectious death among older adults (Carbon, 1993; Feikin et al., 2000; Janssens, 2005; Yende et al., 2005). Notably, the risk for pneumonia increases with the number of underlying comorbidities and inflammatory status (Fabbri et al., 2015; Janssens, 2005; Pelton et al., 2019; St Sauver et al., 2015); Around 90% of older adults who become hospitalized with pneumonia have two or more preexisting comorbid conditions (Barbé‐Tuana et al., 2020; Yende et al., 2005).

Alveolar macrophages (AM) are the most abundant resident immune cells within the airway, where they act as a sentinel cell and are the first to encounter and engage with pathogens (Allard et al., 2018; Hamidzadeh et al., 2017; Hussell & Bell, 2014). Additionally, they produce the majority of cytokines within the lung at the onset of infection and are responsible for recruiting polymorphonuclear cells (PMNs) and monocytes to the site of infection (Allard et al., 2018; Beck‐Schimmer et al., 2005; Gupta et al., 1996; Hamidzadeh et al., 2017; Hussell & Bell, 2014). Thus, defects in the ability of AM to respond to a pathogen can have considerable adverse consequences for the host (Ferrucci et al., 2005; Marcos‐Pérez et al., 2020; O'Mahony et al., 1998; Varadhan et al., 2014). Age‐related defects reported in AM include increased oxidative stress, higher baseline levels of NFκB activation, and altered polarization (Allard et al., 2018; Boyd et al., 2012; Canan et al., 2014; Hinojosa et al., 2009, 2014; Lafuse et al., 2019; Shivshankar et al., 2011; Wang et al., 2017; Wong et al., 2017). We have also reported that AM from aged animals had disrupted TLR signaling, muted p38 MAPK signaling, and lower overall NFκB activation following exposure to infectious stimuli (Hinojosa et al., 2009). The suppression of these cellular activation pathways contributed to the diminished cytokine production by these cells following infection (Boehmer et al., 2004; Boyd et al., 2012; Hinojosa et al., 2009, 2014; Knapp et al., 2003; Metcalf et al., 2015). Up to this point, demonstrations of the impact of aging on susceptibility to airway infections have shown that AM become less functional with age and have defects that accumulate and contribute to an inability to resolve inflammation at the site of infection (Beck‐Schimmer et al., 2005; Gupta et al., 1996; Knapp et al., 2003; Wong et al., 2017).

We have previously demonstrated that TNF contributes to age‐dependent macrophage dysfunction (ADMD) and the enhanced susceptibility of aged animals to infection. Macrophages treated with recombinant TNF and challenged with *Spn* had diminished cytokine production and bacterial killing ability (Hinojosa et al., 2009, 2014; Shivshankar et al., 2011; Thevaranjan et al., 2017). Additionally, infusion of young mice with age‐relevant levels of TNF via an osmotic pump increased the expression of laminin receptor (LR), polymeric immunoglobulin receptor (pIgR), and platelet‐activating factor receptor (PAFr), in a manner similar to what has been observed in aged mice (Shivshankar et al., 2011). These host proteins are co‐opted by *Streptococcus pneumoniae* (*Spn*, the pneumococcus), the leading cause of community‐acquired pneumonia, and other respiratory tract pathogens to attach to cells, and their upregulation has been shown to increase permissiveness for infection (Cundell et al., 1995; Hinojosa et al., 2009; Shivshankar et al., 2011; Swords et al., 2000; Weiser et al., 1998). Crucially, aged mice from a TNF KO background were more resistant to *Spn* infection. What is more, macrophages isolated from aged TNF KO mice did not display the ADMD phenotype (Hinojosa et al., 2009, 2014; Puchta et al., 2016; Thevaranjan et al., 2017). Importantly, the specific mechanism as to how TNF mediates ADMD remained to be determined. Herein, and to address this lapse in knowledge, we have examined the cell signaling changes that occur within aged AM in a TNF‐induced manner. Our results implicate a pan‐suppressive effect of TNF, via upregulation of homeostatic regulators, on the ability of AM to become activated in response to the bacterium.

## 2 MATERIALS AND METHODS

### 2.1 Mice and bacteria

For experiments requiring live animals, young (3–6 months) and aged (18–24 months) C57BL/6 mice of both sexes were obtained from the National Institute on Aging (NIA) or raised at either UAB or McMaster University's animal facilities. All animal experiments were performed in compliance with approved Institutional Animal Care and Use Committee protocol at the University of Alabama at Birmingham and McMaster University. Infection experiments were performed using *S. pneumoniae* serotype 4, strain TIGR4 (Tettelin et al., 2001). Animal challenge experiments were performed using forced aspiration by pipetting 100 μL of 10^5^ colony forming units (CFU) of TIGR4 into the mouth of anesthetized mice hanging by their incisors and covering their nares (Shivshankar et al., 2011). Ethanol‐killed bacteria were prepared by treating *S. pneumoniae* with 70% ethanol for 15 min, which was centrifuged and resuspended in phosphate‐buffered saline (PBS). Inoculation with ethanol‐killed TIGR4 was also done intratracheally but with 10^8^ CFU equivalence of ethanol‐killed TIGR4. To reduce unnecessary use of live animals, the number of mice used for each experiment was determined by power analyses following the completion of the first replicate. Cohort size was based on having sufficient confidence in power of the statistical test applied.

### 2.2 Detection of bacterial burden

Blood was collected from tail bleeds. Lungs, spleens, and hearts of infected animals were extracted into 1 mL of PBS and homogenized. Bacteria were enumerated by the serial dilutions of blood or organ homogenates, plating on blood agar, and enumeration of colony counts following overnight incubation at 37°C in 5% CO_2_. Our limit of detection is denoted on the *x*‐axis of graphs with a less than or equal value (≤). Values with CFU number of being equal to limit of detection are done for graphical purposes so that the CFU that are below the limit of detection can still be represented on the logarithmic graph.

### 2.3 Histology

For BALF analysis, samples were centrifuged onto cytospin slides at 300 *g* for 7 min using the Shandon Cytospin 4 (Thermo Fisher Scientific). Slides were stained according to the Hema 3 staining system protocol (Fisher Healthcare) and imaged using a Leica LMD6 with a DFC450C‐5‐megapixel RGB CCD camera (Leica Biosystems, Buffalo Grove, IL). The cell types were quantified using ImageJ software with the Cell Counter plugin.

### 2.4 Adoptive transfer

Alveolar macrophages in mice were depleted by intratracheal instillation of (100 μL) clodronate or control liposome suspension into the lungs (dosage of 3.75 mg/mL). Standard Macrophage Depletion Kit containing Clodrosome® and Encapsome® control liposomes were purchased from Encapsula NanoSciences (CLD‐8901). The depletion of macrophages was validated by collecting bronchioalveolar lavage fluid and performing cytospins. The next day, 2.5 × 10^5^ AM from donor mice were reconstituted into the lungs intratracheally of recipient mice. Donor primary AM were isolated by bronchoalveolar lavage performed on euthanized mice with 3 mL of PBS with 1 mM EDTA. The lavage was centrifuged at 300 *g* for 10 min and resuspended in 100 μL PBS that was intratracheal instilled into the lungs of recipient mice.

### 2.5 Phosphorylation array and functional test of AM

Mice were asphyxiated with isoflurane in a glass jar, and bronchoalveolar lavage was performed using 3 mL of ice‐cold PBS. Bronchoalveolar lavage fluid (BALF) was centrifuged, and pelleted cells were then suspended in Dulbecco's modified Eagle medium with heat‐inactivated FBS, 10 mM MEM Non‐essential amino acids, 50 mM 2‐mercaptoethanol, and 1 M HEPES and counted with a hemocytometer and seeded in a tissue culture plate. Following 1 h of adherence at 37°C in 5% CO_2_, nonadherent cells were removed by gently washing two times. For the phosphorylation array, cells were infected with *Spn* in DMEM at an MOI of 25. The plates were centrifuged to synchronize infection at 300 g for 5 min. Cells were incubated at 37°C in 5% CO_2_ for 15 min. The supernatant was removed, and cells were lysed with RIPA buffer containing phosphatase and protease inhibitors and scraped from the plate. The samples were agitated for 30 min on a rocker and then spun down at 20,000 *g* for 20 min to collect the protein supernatant. BCA was done to quantify the amount of protein present. The protein was then loaded onto the phosphoarray kit from RayBiotech (AAH‐MAPK‐1‐8). Phagocytosis was done using an Abcam Phagocytosis Assay Kit (ab234053). Macrophage killing assay was performed as previously described (Thevaranjan et al., 2017).

### 2.6 ELISAs and immunoblots

The detection of inflammatory markers was done using MPO (DY3667), TNF (DY410), KC (DY453), and IL‐6 (DY406) ELISA kits from R&D Systems Inc. (Minneapolis, MN, USA). Albumin ELISA was bought from Bethyl Laboratories (E99‐134). Immunoblots were performed using standard methods. Protein concentrations were measured by BCA assay (Bio‐Rad Laboratories, Hercules, CA, USA) and normalized to the same concentration. Lysates were boiled for 10 min at 95°C with NuPAGE LDS sample buffer, and equal amounts were added to each well and run on 10% Mini‐PROTEAN® TGX™ Precast Gels (Bio‐Rad). Proteins were then transferred onto a 0.2 μm Nitrocellulose membrane (Bio‐Rad, 1704158). Membrane blocking was done with 10% BSA followed by probe IgGs specific for *Dusp1* (Cell Signaling, 35217S, 1:500), Ptprs (Proteintech, 13008‐1‐AP, 1:500), or Actin (Abcam, Ab8226, 1:10,000) overnight at 4°C. Bound antibody detected using secondary IgG conjugated with horseradish‐peroxided (Abcam, ab6721) followed by ECL chemiluminescence (Pierce™ ECL western blotting substrate, PI32209) and imaged using the Bio‐Rad ChemiDoc™ XRS^+^ (Bio‐Rad). Densitometry was performed using ImageJ software.

### 2.7 RNA sequencing

Lungs from young and aged mice where cells were sent off to Northwestern for digestion and cell sorting, AM were >95% purity. Macrophages were collected from both a C57Bl/6 and TNF KO background of mice. All mice were female and aged to 20 months of age. RNA was isolated using RNeasy Plus Mini kits (Qiagen) followed by mRNA magnetic enrichment using NEBNext kit (New England Biolabs). Libraries were then sequenced on NextSeq 500 instrument (Illumina) at 75‐bp length, single reads with an average reading depth exceeding 3 × 10^6^ per sample. Reads were demultiplexed using bcl2fastq, trimmed, aligned to reference genome mm10 using TopHat2, and quality was assessed with FastQC (Anders et al., 2015; Kim et al., 2013). Aligned reads were mapped to genes using HTseq with an Ensembl annotation. The trimmed mean of *M*‐values (TMM) normalization was used to normalize the dataset prior to differential expression analysis (Robinson & Oshlack, 2010). Differentially expressed genes (DEGs) were identified using the DESeq2 package on R studio (Love et al., 2014). Differentially expressed genes were analyzed using the web‐based pathway analysis tool QIAGEN IPA (www.ingenuity.com) to identify pathways altered during aging and due to TNF exposure with log2 fold change of 1.5 and adjusted *p*‐value cutoff set to 1e‐3 for independent filtering (Krämer et al., 2014). PCA and IPA plots were made on R Studio using the ggplot2 package (Wickham, 2016). The RNAseq files are publicly available on GEOSOURCE.

### 2.8 RNA quantification

Gene levels of Ptprs, Dusp1, and A20 were detected using qPCR. RNA was collected from AM or J774.1 cells using Trizol (Thermo Fisher 15596026) chloroform extraction. cDNA conversion was done using Applied Biosystems™ High‐Capacity cDNA Reverse Transcription Kit (4368814). RT qPCR was done with the QuantiTect SYBR Green PCR Kit (204143) and was read on a Bio‐Rad CFX Opus Real‐Time System. The values were normalized according to the delta delta CT method with two different housekeeping genes (*Tbp* and *RPL13a*). In doing so, we are able to normalize the quantity of *Dusp1* and *Ptprs* per each individual sample divided by its expression of housekeeping genes. Each sample is run in triplicate on at least two separate occasions.

### 2.9 Statistical analysis

All results are displayed with error bars indicating the median with interquartile range, unless otherwise indicated. For in vivo experiments, each individual data point represents an individual animal sample with the number of animals denoted in the figure legend. For data with a single independent factor or two groups, we used a Mann–Whitney U test, unless otherwise indicated. For parametric grouped analyses, we used ANOVA followed by the Bonferroni post hoc analysis or Fisher's LSD test. The Bonferroni post hoc analysis was used for multivariate comparisons to correct for multiple comparisons. Fisher's LSD test was used when the ANOVA result was significant and each pairwise comparison stands alone. A Kruskal–Wallis test was used, to compare nonparametric data, followed by Dunn's multiple comparisons test for hypothesis testing due to the low sample size. Asterisks denote the level of significance observed * = *p* ≤ 0.05; *** = *p* ≤ 0.01; *** = *p* ≤ 0.001; **** = *p* ≤ 0.0001. Statistical analysis was performed using PRISM 9 (GraphPad Software: La Jolla Ca).

## 3 RESULTS

### 3.1 Aged mice are more susceptible to pneumococcal disease and have defects in their ability to respond to infectious stimuli

**FIGURE 1:**
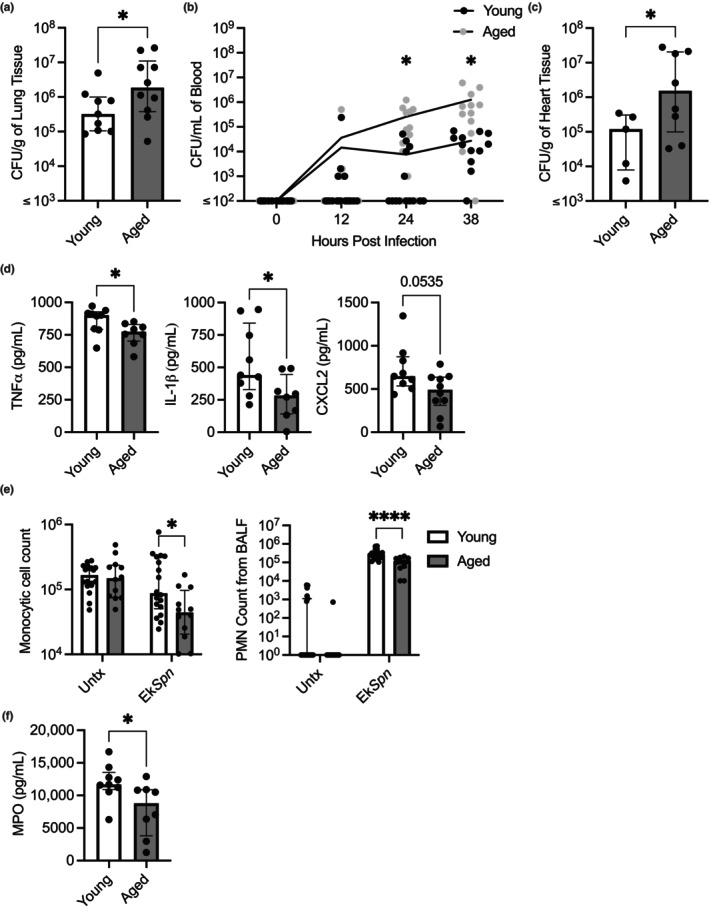
Aged mice are more susceptible to pneumococcal disease and have defects in their ability to respond to infectious stimuli. Young (3–6 months) and aged (18–24 months) C57BL/6 mice were infected intratracheally with 10^5^ CFU of *Spn*. Mice were sacrificed at 24 h postinfection. The bacterial burden in the lung was enumerated from serial dilutions of lung homogenates on blood agar plates (a). Tail bleeds were taken throughout the infection to enumerate bacterial burden within the blood (b). The bacterial burden in the heart was enumerated from serial dilutions of heart homogenates on blood agar plates (c). Young (3–6 months old) and aged mice (18–24 months old) were intratracheally inoculated with 10^8^ CFU equivalents of ethanol killed Spn (Ek*Spn)*. At 8 h postinoculation, mice were sacrificed and bronchoalveolar lavage fluid (BALF) was collected. Levels of TNFα, IL‐1β, and CXCL2 in BALF as measured by ELISA (d). Monocytes and PMNs within BALF were attached to slides by cytocentrifugation, stained, and enumerated by nuclear morphology analysis (e). Myeloperoxidase levels in BALF was measured (f). Statistical significance was calculated using a nonparametric Mann–Whitney U test (a, c, d, & f), a repeated measures two‐way ANOVA with Fisher's LSD post hoc test (b), a two‐way ANOVA with Bonferroni multiple comparisons post hoc test (e). The data are presented as median with interquartile range (IQR); *p ≤ 0.05; ****p ≤ 0.0001. Each data point represents an individual mouse. Graphs with ≤ on *x*‐axis indicate limit of detection.

Consistent with the greater susceptibility to infection observed in older human adults, aged mice (18–24 months) intratracheally challenged with *Spn* experienced greater bacterial burden than their younger counterparts (3–6 months). One day after the challenge, lungs isolated from infected aged mice had a 15‐fold higher bacterial burden than young mice (Figure 1a). Older humans are also far more likely to develop invasive pneumococcal disease, that is, bacteremia, during pneumonia than younger adults (Kyaw et al., 2005; Loeb, 2004; Pelton et al., 2019). Consistent with this, aged mice also had greater levels of *Spn* in their blood across time points than young controls, culminating in a nearly 40‐fold median difference at 38 h postinfection (Figure 1b). Once in the bloodstream, *Spn* can disseminate systemically, invade tissues, and cause long‐lasting organ damage (Brown et al., 2014; Eurich et al., 2017; Kruckow et al., 2023; Musher et al., 2007, 2019; Reyes et al., 2017). We observed over a 10‐fold higher median burden of *Spn* within the heart tissue of aged mice compared to young mice (Figure 1c). Thus, aged mice rapidly developed the most severe forms of pneumonia and invasive pneumococcal disease, along with its complications, making them suitable models to study age‐related defects in susceptibility.

To better understand why aged mice are more susceptible to *Spn*, we tested their ability to mount an early immune response to an infectious challenge. Importantly, the extreme differences in bacterial burden and the inability of aged mice to constrain bacterial replication seemed likely to impact the scale of their inflammatory response even at early time points. Therefore, we instead challenged mice intratracheally using ethanol‐killed *Spn* (Ek*Spn*), which could not replicate, to ensure that all mice received an equal pro‐inflammatory signal. Following the forced aspiration of *EkSpn*, we collected bronchoalveolar lavage fluid (BALF) and measured the levels of pro‐inflammatory cytokines and immune cells present. Aged mice had blunted production of TNF and IL‐1β (Figure 1d). Additionally, aged mice had trending but not significantly decreased levels of the chemokine macrophage inflammatory protein 2 (MIP‐2, also known as CXCL2), which is a PMN chemoattractant (Figure 1d; Gupta et al., 1996; Schmal et al., 1996; Standiford et al., 1996). Consistent with the latter, aged mice had significant reductions in the number of PMNs as well as monocytes in the airway following the challenge (Figure 1e). Notably, PMN numbers in the airway corresponded with decreased neutrophil activity in the airway as measured by myeloperoxidase (MPO; Figure 1f). What is more, measured levels of MPO in the airway were greater for young versus aged mice when values from mice with similar numbers of PMN in the airway were considered (*n* = 4, samples with higher than 20,000 neutrophils, *p* = 0.0401), suggesting that the aged PMNs are not as functional. Based on these results, we purport that unchecked bacterial replication in the lungs of aged mice following aspiration results from a muted inflammatory response and decreased or potentially delayed immune cell recruitment as a consequence of unresponsive AM. These results also suggest that age‐related defects in cell types other than AM, including PMNs, are most likely also contributing to the increased susceptibility to infection.

### 3.2 Age‐dependent macrophage dysfunction is sufficient to increase susceptibility to pneumococcal disease

**FIGURE 2:**
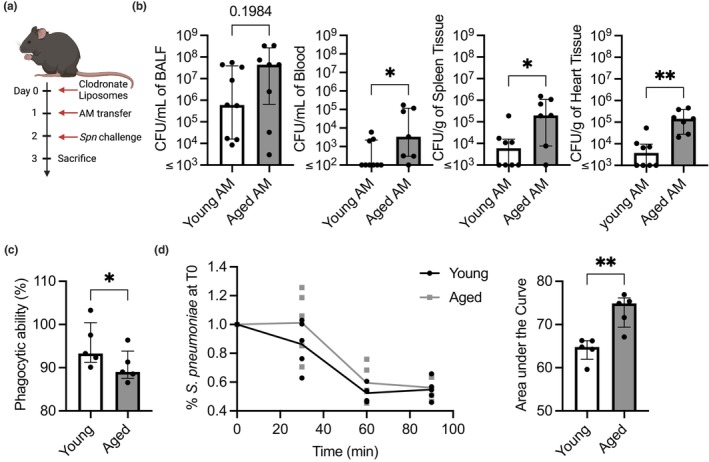
Age‐dependent macrophage dysfunction is sufficient to increase susceptibility to pneumococcal disease. Young mice depleted of their own alveolar macrophages using clodronate liposomes were adoptively transferred with AM from young or aged mice and subsequently challenged intratracheally with 10^5^ CFU of *Spn* (a). Following 1 day, the bacterial burden was enumerated from BALF, blood, spleen, and heart homogenates (b). AM isolated from BALF of young and aged mice were used in functional assays testing their ability to phagocytize prelabeled zymosan particles (c), and an ex vivo macrophage killing assay of *Spn* that was quantified by the area under the curve (d) to compare the functionality of young versus aged macrophages. Statistical significance was calculated using a Mann–Whitney U test (b, c, & d). The data are presented as median with IQR; *p ≤ 0.05; **p ≤ 0.01. Each data point represents an individual mouse (b) or well (c & d). Graphs with ≤ on *x*‐axis indicate limit of detection.

AM have been shown to become less functional with age, but the degree to which this by itself impacts infection risk has not been specifically tested (Beck‐Schimmer et al., 2005; Canan et al., 2014; Knapp et al., 2003; Lafuse et al., 2019; Wong et al., 2017). To discern this, we adoptively transferred AM from either young or aged mice into the lungs of young mice that had been treated prior with clodronate liposomes to deplete resident macrophages and subsequently experimentally challenged these mice with live *Spn* (Figures S1a and S2a). Although bacterial load in the BALF only trended upward 1 day post‐challenge, we observed a stark loss of containment of the infection and a 40‐fold difference in the median number of bacteria in the blood as well as nearly 20‐fold differences in the spleen and heart, compared to mice that received AM from young donors (Figure 2b). Notably, when AM from young mice were instilled into aged mice, we saw no statistically significant differences in burden when compared to aged mice that received AM from aged mice (Figure S1b). Thus, and as would be expected, other age‐related factors are also contributing to the enhanced susceptibility of aged mice to infection. To further clarify the consequences of ADMD on susceptibility to infection, we isolated AM from young and aged mice and performed ex vivo functional analysis. This revealed that AM from aged mice were attenuated in their capacity to phagocytize and kill pneumococci (Figure 2c,d). We conclude that defects present in aged AM are sufficient to increase disease severity, and their dysfunction directly contributes to a loss of airway containment of *Spn* infection. Although, as young AM transferred into an aged host are not sufficient to lower susceptibility to infection, we conclude that other age‐related defects are also contributing to the increased susceptibility to infection.

### 3.3 Chronic exposure to TNF contributes to the hyporesponsiveness of aged AM to Sp*n*

**FIGURE 3:**
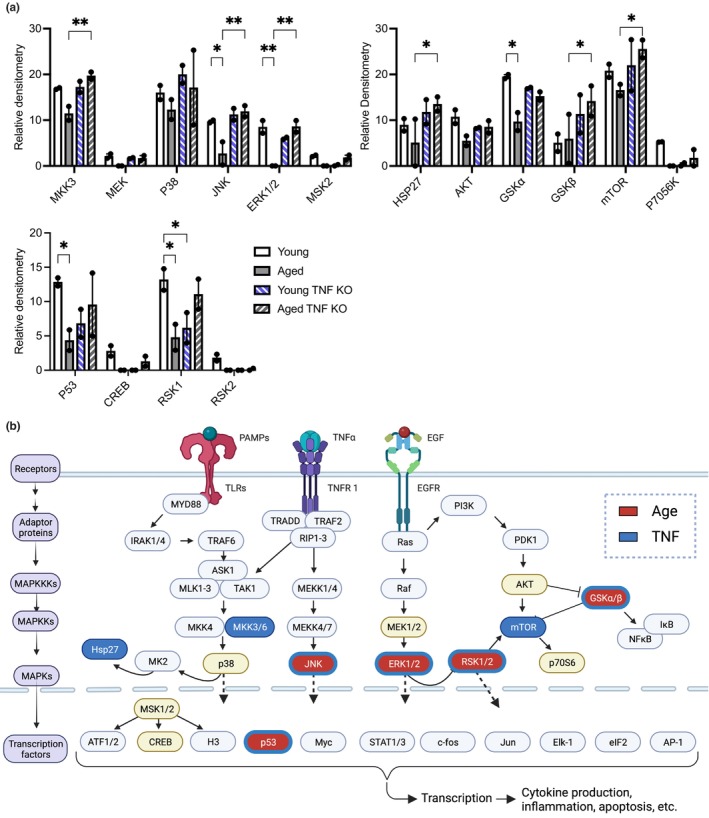
Phosphokinase signaling is starkly suppressed in aged AM. AM were collected from young and aged WT and TNF KO mice and inoculated with *Spn* at an MOI of 25 for 15 min. Purified protein from these samples was used in a phosphorylation array of the MAPK pathway (a) with results overlaid on the MAPK pathway (b). Any protein designated by a colored oval was tested as part of the phosphorylation array, with items in red indicating significant changes due to age and blue indicating alterations due to TNF. Statistical significance was calculated using a two‐way ANOVA with each comparison standing alone using a Fisher's LSD test. The data are presented as median with interquartile range (IQR); *p ≤ 0.05; **p ≤ 0.01. Each individual point represents the average of two technical replicates on the phosphorylation array per biological sample.

To gain an understanding of the full impact of both aging and TNF on macrophage function and TNF‐mediated MAPK signaling suppression, we examined the activation status of AM from young and aged, in both wild‐type and TNF KO mice backgrounds, using phosphorylation arrays containing antibodies against representative MAPK signaling proteins. In brief, we exposed both wild‐type and TNF KO AM from young and aged mice to Ek*Spn* and assessed their phosphorylation status using an antibody array recognizing the phosphorylated versions of 16 MAPK signaling proteins. We observed significant age‐associated reductions in the phosphorylation status of more than a quarter of the kinase targets assayed (P53, ERK1/2, RSK1, JNK, and GSKα) for wild‐type mice (Figure 3a). Strikingly, most of these reductions could be directly attributed to TNF, as there were no age‐related differences observed between young and aged mice in the TNF KO background, and the activation levels seen in aged TNF KO were equivalent to that of young wild‐type mice (p53, ERK1/2, JNK, & GSKα; Figure 3a). We also observed differences due to the presence of TNF regardless of age, including MKK3, HSP27, GSKβ, mTOR, RSK1, and JNK (Figure 3a). We overlaid the results on a diagram illustrating the primary MAPK signaling cascades (Figure 3b). From this, we could infer that there were global alterations in MAPK signaling with diminished phosphorylation at the level of MAPKKs, MAPKs, transcription factors, and effector molecules and that, in some instances, multiple levels of individual cascades were suppressed (e.g., MKK3, p38, hsp27, and msk1/2).

### 3.4 TNF impacts the transcriptome of AM but not as much as normal aging

**FIGURE 4:**
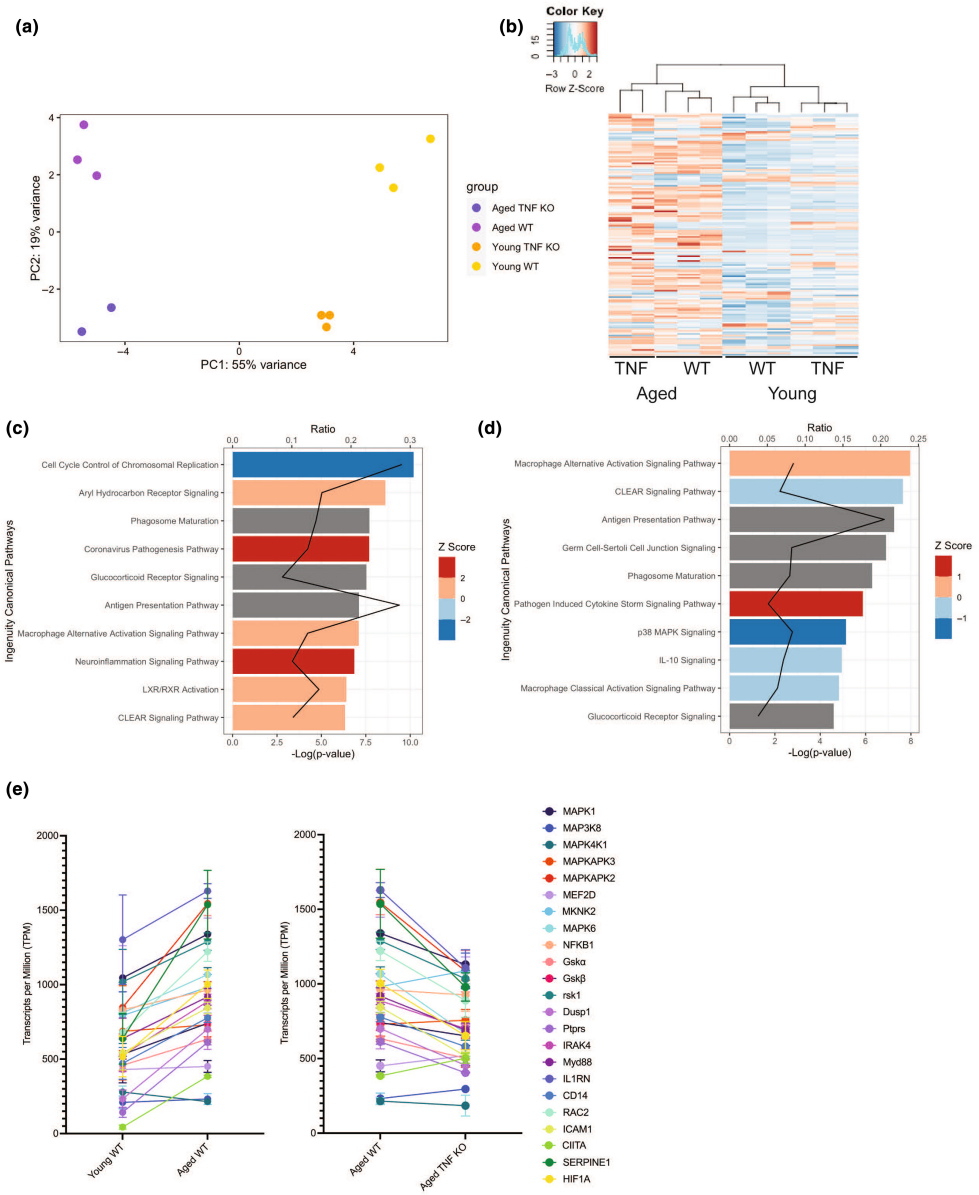
AM have age and TNF‐dependent differences in gene expression. RNA was purified from AM collected from young (3–6 months old) and aged mice (18–24 months old) in both wild‐type C57BL/6 and TNF KO mice. Alterations in gene expression due to age and TNF expression were visualized using a PCA plot (a) and a dendrogram (b). IPA was used to show the 10 most highly scoring canonical pathways (according to *p* value) altered in expression due to aging (c) and due to genotype (d) by using comparisons of aged WT versus young WT and Aged TNF KO vs Aged WT, respectively. The red line represents the ratio of the number of differentially expressed genes within a pathway divided by the total number of genes within the pathway. Bars represent the *p*‐value for each pathway expressed as −1 times the log of the *p*‐value, with the color of the bar indicating the *Z* score. Relative expression of various members of the MAPK signaling cascade visualized in transcripts per million by group (e).

In parallel, gene expression profiles of AM isolated from similar cohorts of mice were obtained using RNA‐seq. Principal component analyses of all genes in the transcriptomic dataset found that each experimental cohort clustered separately, whereas biological replicates within each cohort clustered together (Figure 4a), indicating generally reproducible differences in gene expression profiles across AM from different mice. Principle component (PC) 1, which composed 55% of the variance among the dataset, matched with the distinct grouping of the mice by age, whereas PC2, which accounted for 19% of the variance, corresponded to the TNF status of the mice. Dendrogram analysis of the gene expression profiles confirmed this separation by grouping samples by two major branches containing young and aged mice with the genetic background grouping independently within the separate age groups of the top 150 variable genes (Figure 4b). Thus, the impact of advanced age on AM gene expression is more significant than TNF deficiency alone. A list of all the genes with differential expression due to aging is provided in Table S1.

We used Ingenuity Pathway Analysis (IPA) for data analysis and identified global differences in biological pathway expression that depended on TNF status (Figure 4c, Table S2) as well as on TNF status and aging (Figure 4d, Table S3; Krämer et al., 2014). Among the most prominent differences between young and aged mice were those for genes involved in cell cycle and proliferation, inflammatory signaling, and antigen presentation pathways. The most prominent differences between aged WT and aged TNF KO mice were primarily among inflammatory signaling pathways, including increased macrophage alternative activation, decreased macrophage classical activation signaling, and MAPK signaling. Both comparisons showed alterations in CLEAR (coordinated lysosomal expression and regulation) signaling, phagosome maturation, glucocorticoid receptor signaling, and macrophage alternative activation. The transcript alterations in the MAPK signaling pathway due to age and TNF are illustrated in a graph of the relative expression of various MAPK signaling members in transcripts per million (Figure 4e). Interestingly, the differences in MAPK signaling contributions from age and TNF were consistent with our prior observations using the phosphorylation arrays. The transcripts from aged mice had higher expression of various MAPK members than young mice, but the aged TNF KO samples had levels that were more similar to those of young mice.

### 3.5 The phosphatases *Dusp1* and *Ptprs* are upregulated in a TNF‐dependent manner during aging

**FIGURE 5:**
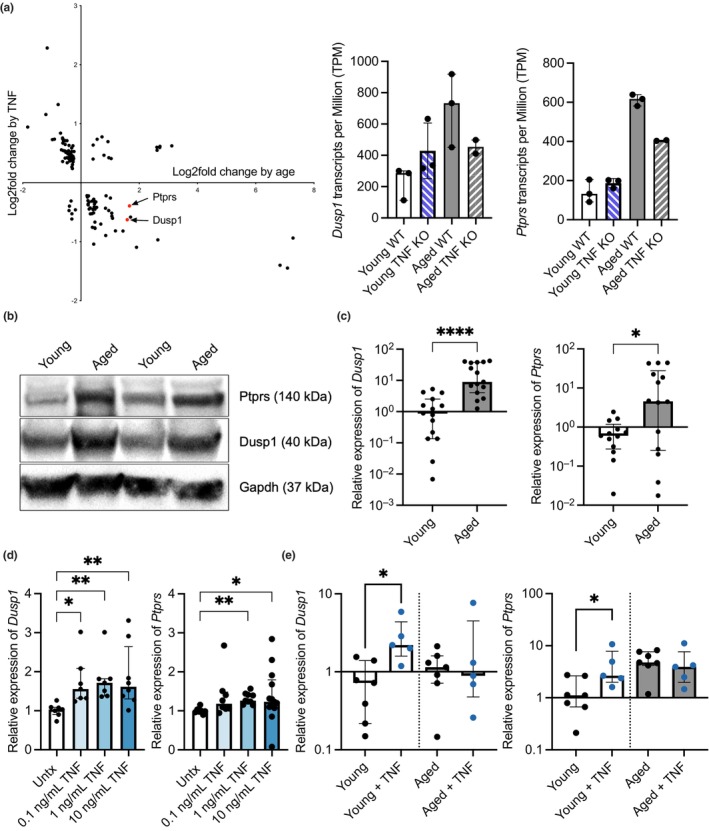
Key negative regulators are upregulated in a TNF‐dependent manner with age. Identification of *Dusp1* and *Ptprs* in DEGs as a result of TNF status and age from the transcriptomic dataset (a). Expression levels of negative regulators *Dusp1* and *Ptprs* from alveolar macrophages are shown in Transcripts per Million (a), protein levels of Dusp1 and Ptprs in whole lung cell lysates via western blots (b), and in isolated AM by qPCR (c). Levels of negative regulators *Dusp1* and *Ptprs* in J774.1 cells treated with various levels of TNF via qPCR (d). Levels of negative regulators *Dusp1* and *Ptprs* in AM from young and aged mice collected and exposed to TNF ex vivo (e). Statistical test was done using either a Kruskal–Wallis (d & e) or a Mann–Whitney test (c). The data are presented as median with interquartile range (IQR); *p ≤ 0.05; **p ≤ 0.001; ****p ≤ 0.0001. Each data point represents an individual gene (a), mouse (b & c), or well (e).

From the transcriptome dataset, we identified two negative homeostatic regulators of MAPK signaling pathways, dual specificity phosphatase 1 (*Dusp1*) and protein tyrosine phosphatase receptor type S (*Ptprs*), which were upregulated in AM from aged WT mice versus AM from young WT mice, but not aged TNF KO mice (Figure 5a). Both negative regulators are members of the protein tyrosine phosphatase (PTP) superfamily and act as inhibitors of MAPK signaling (St‐Denis et al., 2016). *Dusp1*, also known as mitogen‐activated protein kinase phosphatase‐1 (MKP‐1), has been shown to have an important role in regulating inflammation by inhibiting MAPK activation via dephosphorylating both the p38 subunit of MAP kinase and ERK2, among other targets (Chi et al., 2006; Chu et al., 1996; Franklin & Kraft, 1997; Hammer et al., 2006; Salojin et al., 2006; Sun et al., 1993; Zhao et al., 2005). *Ptprs*, a leukocyte common antigen‐related (LAR) receptor‐type phosphatase, has been shown to inhibit the activation of dendritic cells and dephosphorylate various signaling molecules, including STAT3, EGFR, AKT, SRC, and ERK (Bunin et al., 2015; Davis et al., 2018, 2018, 2019; den Hertog et al., 2004; Gong et al., 2021; Wang et al., 2015). These results were corroborated by immunoblot using whole lung cell lysates from young and aged mice (Figure 5b), as well as qRT‐PCR of isolated RNA taken from AM collected from individual young and aged mice (Figure 5c). As these genes were not elevated in the transcripts from the AM of aged mice with a TNF KO background, we wanted to test whether these genes were TNF regulated. TNF treatment increased the expression of both *Dusp1* and *Ptprs* in J774.1 cells (Figure 5d), albeit only at higher concentrations for the latter. In contrast, we observed a meaningful increase in *Dusp1* and *Ptprs* gene expression levels in AM from young mice administered TNF ex vivo, but no difference in aged mice (Figure 5e). We interpret these results as meaning that *Dusp1* levels are directly responsive to TNF, whereas an intermediary signaling molecule may be required for *Ptprs* gene expression. Moreover, as exogenous TNF treatment of AM from aged animals did not further increase *Dusp1* and *Ptprs* gene expression levels, a saturated response to TNF may already be in place.

### 3.6 *Dusp1* and *Ptprs* have suppressive effects on macrophage function, and anti‐TNF can alleviate this during aging

**FIGURE 6:**
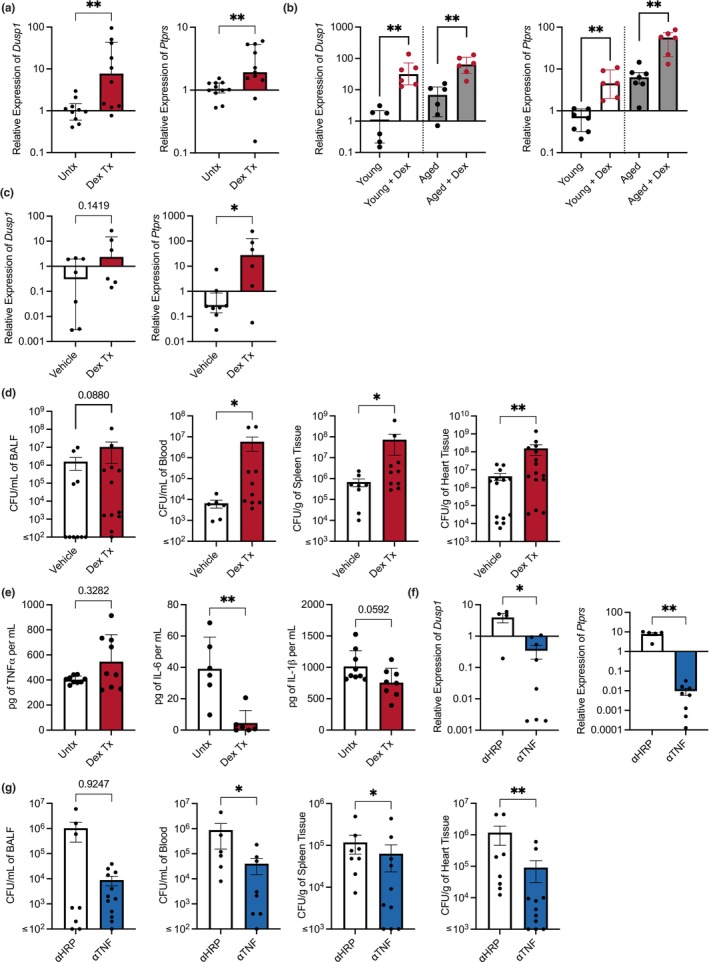
Manipulating the levels of negative regulators alters responses to infection. RNA was isolated from J774.1 macrophages that were treated with dexamethasone, and the levels of *Dusp1* and *Ptprs* were quantified with qPCR (a). The levels of *Dusp1* and *Ptprs* were quantified with qPCR on alveolar macrophages from young and aged mice treated with dexamethasone ex vivo (b) as well as in vivo (c). We quantified the levels of pro‐inflammatory cytokines produced by J774.1 cells treated with dexamethasone overnight and then challenged with Ek*Spn* (e). Young (3–6 months) C57BL/6 mice were treated with dexamethasone and infected intratracheally with 10^5^ CFU of *Spn*. Bacterial burden 24 h postinfection, in the BALF, blood, spleen, and heart was enumerated (d). C57BL/6 mice were treated every other day with anti‐TNF or anti‐HRP control antibodies for 2 weeks before being infected intratracheally with 10^5^ CFU of *Spn*. Mice were sacrificed 24 h postinfection, the heart and spleens were harvested, and blood and BALF were collected. RNA was isolated from alveolar macrophages from mice that were treated with anti‐TNF or anti‐HRP control antibodies and were quantified for levels of negative regulators (f). Bacterial burden in mice treated with anti‐TNF was enumerated in the BALF, blood, spleen, and heart (g). The data are presented as median with interquartile range (IQR); *p ≤ 0.05; **p ≤ 0.01. Each data point represents an individual mouse (b, d, e, f, & g) or well (a & c). Graphs with ≤ on *x*‐axis indicate limit of detection.

The suppressive effects of Dusp1 on cell signaling are documented as being potent and considered to be the molecular mechanism behind the anti‐inflammatory and immunosuppressive effects of corticosteroids (Abraham et al., 2006; Chen et al., 2002; Fürst et al., 2007; Kassel et al., 2001; Manetsch et al., 2012a; Reddy et al., 2009). Accordingly, the promoter region of *Dusp1* contains multiple glucocorticoid response elements, and glucocorticoid treatment has been shown to increase the expression of *Dusp1* (Abraham et al., 2006; Shipp et al., 2010). Although direct evidence of *Ptprs* upregulation upon glucocorticoid treatment has not yet been demonstrated, our analysis using the publicly available ENCODE Transcription Factor Targets dataset revealed that *Ptprs* does have glucocorticoid response elements within its promoter (Rouillard et al., 2016). Congruently, dexamethasone treatment increased the expression of *Dusp1* and *Ptprs* in not only J774A.1‐treated cells in vitro (Figure 6a) but also AM isolated from young mice treated ex vivo (Figure 6b), and AM from young mice administered the steroid (Figure 6c). Moreover, dexamethasone treatment recapitulated the ADMD phenotype, as treated J774A.1 cells produced less pro‐inflammatory cytokines (Figure 6d), and dexamethasone‐treated young mice had greater susceptibility to *Spn* infection (Figure 6e). Further linking age‐associated levels of TNF to heightened levels of these cell signaling suppressors and greater susceptibility to infection, we observed that the treatment of aged mice with anti‐TNF lowered *Dusp1* expression eightfold and *Ptprs* by 50‐fold (Figure 6f). What is more, aged mice treated with anti‐TNF antibody prior to infection experienced less severe disease, evidenced by the decreased bacterial burden in the blood, spleen, and heart compared to mice treated with anti‐HRP isotype control antibody (Figure 6g). Additionally, we validated that the anti‐TNF treatment lowered the BAL levels of TNF (Figure S2a). Thus, ADMD closely parallels the immune suppressive state that develops with glucocorticoid treatment, and the upregulation of *Dusp1* and *Ptprs* in AM due to TNF is a molecular explanation for this.

## 4 DISCUSSION

Chronic inflammation and its failure to resolve are central to the development and complications of several age‐related conditions. Specifically, high plasma levels of TNF have been associated with the pathophysiological mechanisms underlying most aged‐related morbidities and diseases (Bae et al., 2017; Beutler et al., 1985; Bruunsgaard et al., 1999, 2003; Fillit et al., 1991; Firestein & McInnes, 2017; Hubbard et al., 2009; McInnes & Schett, 2007; Van Deventer, 1997). Accordingly, chronic low‐grade inflammation is associated with multi‐morbidity, functional disability, and increased mortality in older adults (Bruunsgaard et al., 1999, 2003; Cohen et al., 1997; Fabbri et al., 2015; Harris et al., 1999; Mooradian et al., 1991; Varadhan et al., 2014; Volpato et al., 2001). Chronic inflammation contributes not only to the development and exacerbation of multiple noncommunicable diseases but also to susceptibility to infection. For example, elevated TNF, IL‐6, and CRP serum levels predispose individuals to community‐acquired pneumonia (CAP) and greater disease severity (Kyaw et al., 2005; Loeb, 2004; Pelton et al., 2019; Yende et al., 2005). In accordance with this, we observed that aged mice had a considerably greater bacterial burden in their lungs and had greater levels of *Spn* in their blood across time points than young controls. This is highly similar to the human condition, in which older humans are also far more likely to develop invasive pneumococcal disease, that is, bacteremia, during pneumonia than younger adults (Kyaw et al., 2005; Loeb, 2004; Pelton et al., 2019). Previous studies from our group have indicated that ADMD contributes significantly to this susceptibility. Along such lines, other chronic inflammatory diseases, such as uncontrolled HIV infection and COPD, induce macrophage dysregulation that includes impaired apoptosis, reduced induction of ROS, and a decline in bacterial killing ability (Bewley et al., 2016). Thus, chronic inflammation caused by aging or disease and associated with increased TNF induces AM dysfunction, contributing to the increased susceptibility to the infection of aged animals and people.

We observed that aged mice had a suppressed response to inflammatory stimuli with muted production of the essential early‐response pro‐inflammatory cytokines, TNF and IL‐1, that are produced primarily by AM in response to *Spn* (Losa García et al., 1999; O'Brien David et al., 1999; Rijneveld et al., 2001; Ulich et al., 1991). Additionally, aged mice had decreased levels of the chemokine macrophage inflammatory protein 2 (MIP‐2, also known as CXCL2), which is produced in the lungs by both AM and alveolar epithelial cells to stimulate and recruit PMNs into the infected airspaces (Gupta et al., 1996; Pittet et al., 2011; Schmal et al., 1996; Standiford et al., 1996). In turn, this result helps explain why aged mice challenged with Ek*Spn* had fewer PMNs infiltrating into their lungs postchallenge. Importantly, a number of studies indicate that neutrophil function is also altered with advanced age (Adrover et al., 2016; Van Avondt et al., 2023). Supportive evidence for this was observed in the levels of MPO detected when examining mice with equal numbers of infiltrated neutrophils. Thus, even though the adoptive transfer of aged AM was sufficient to enhance the susceptibility of young mice to infection, multiple other defects in host immunity are contributing to the enhanced susceptibility of aged animals.

From our adoptive transfer experiment, we observed that properties intrinsic to aged macrophages were sufficient to increase susceptibility to infection regardless of the youth of the surrounding host, suggesting that the “young” microenvironment could not overcome preset dysfunction in cells. These effects may be due to inherent defects of aged AM arising from a lifetime of replication and inflammatory stimuli exposures. Alternatively, this may be due to transient but prolonged effects due to the continual modification of AM behavior with residual proteins left over from the aged environment, such as elevated *Dusp1* and *Ptprs*. We also observed that young AM transferred into aged mice could not overcome the microenvironment, and there were no differences in burden between aged mice that received young AM compared to those that received aged AM. This result could be explained by the aforementioned dysfunction in other cell types, such as PMNs. Of note, in our Ek*Spn*‐challenged mice, we saw decreased numbers of monocytic cells in the airway of aged mice compared to untreated controls. Additionally, It has recently been shown that AM undergo apoptosis to eliminate *Spn* from the airway (Preston et al., 2019). We do not know whether the apoptotic‐associated bacterial killing is impacted by age, which could alter cytokine production and subsequent recruitment of immune cells. However, this is an interesting point for future studies.

From our transcriptomics analyses, we observed that *Dusp1* and *Ptprs* were upregulated due to aging in a TNF‐dependent manner. Both negative regulators are tyrosine phosphatases, with *Dusp1* having dual specificity for threonine, and serve to shut down kinase‐mediated signaling cascades for various cellular processes limiting inflammation (Seternes et al., 2019). MAPK signaling pathways play an essential role in regulating cellular processes such as the cell cycle, cellular proliferation, and the rapid initiation of macrophage‐mediated inflammatory responses (Yang et al., 2014; Zhang & Liu, 2002). MAPK signaling activation also controls the expression of cytokines (TNF, IL‐1β, IL‐6), chemokines (CXCL1 and CXCL2), and inflammatory mediators production (prostaglandin E_2_, cyclooxygenase‐2, and iNOS; Yang et al., 2014). Notably, the increased expression in *Dusp1* and *Ptprs* has been shown to occur due to many chronic inflammatory diseases, including cancer, rheumatoid arthritis, and ulcerative colitis (Berillo et al., 2022; Davis et al., 2018; Hendriks & Pulido, 2013; Khadir et al., 2018; Liu et al., 2019; Muise et al., 2007; Xu et al., 2015). Yet the deletion of *Dusp1* in animal models enhanced susceptibility to pathogens, including *Mycobacterium tuberculosis*, *Chlamydophila pneumonia, Staphylococcus aureus*, and *Escherichia coli* (Cheung et al., 2009; Frazier et al., 2009; Gräb et al., 2019; Hammer et al., 2010; Kim et al., 2012; Li et al., 2023; Rodriguez et al., 2010; Wang et al., 2007; Zhao et al., 2006). Thus, the negative regulators, *Dusp1* and *Ptprs*, help maintain a level of inflammation essential for the development of an appropriate antimicrobial response and whose perturbation by deletion is too far‐reaching. The induction of these negative regulators can be considered to be an appropriate, purposeful response by the host to limit inflammation and prevent a destructive positive feedback loop that can aggravate or worsen chronic conditions. However, when infection occurs under these circumstances, AM are suppressed in their ability to respond due to the dampening of MAPK pathways and, therefore, unable to control the infection effectively. Thus, these negative regulators have a previously unappreciated role in susceptibility to infection during advanced aging.

Notably, glucocorticoid signaling was identified as one of the top altered pathways in our transcriptome dataset due to both aging and TNF status. The promoter region of *Dusp1* is tightly regulated and contains binding sites for several transcription factors, including a glucocorticoid receptor (Shipp et al., 2010), which was taken advantage of in our study to determine the consequence of its upregulation on the AM response to infection. We found that *Ptprs* was also induced by the glucocorticoid dexamethasone, which makes sense, as according to the ENCODE Transcription Factor Targets dataset, *Ptprs* has glucocorticoid response elements within its promoter (Rouillard et al., 2016). What is more, glucocorticoid treatment on both AM and macrophage‐like cell lines showed increased expression of *Dusp1* and *Ptprs* while simultaneously suppressing the ability to respond to bacteria. Furthermore, young mice pretreated with dexamethasone before infectious challenge with *Spn* had higher expression of negative regulators in AM and worse disease than vehicle‐treated controls. This coincides with data showing that individuals taking corticosteroids, for example, patients with asthma, COPD, rheumatoid arthritis, and other inflammatory conditions, are at an increased risk for developing pneumonia (Calverley et al., 2007; Dixon et al., 2011; Doran et al., 2002a; Ferguson et al., 2008; Kardos et al., 2007; Suissa et al., 2013; Zhang et al., 2013). Importantly, corticosteroid therapy is often recommended in conjunction with antibiotics in patients hospitalized with severe pneumonia, as in this circumstance, the bacteria are eradicated and steroid treatment is warranted for reductions in the need for mechanical ventilation, length of hospital stay, risk of acute respiratory distress syndrome, and all‐cause mortality (Dequin et al., 2023; Meijvis et al., 2011; Remmelts Hilde et al., 2012; Siemieniuk et al., 2015; Torres et al., 2015). Pertinent to this discussion, it is well established that the immunosuppressive therapeutic action of glucocorticoids depends on the activation of *Dusp1* (Abraham et al., 2006; Fürst et al., 2007; Hoppstädter & Ammit, 2019; Kassel et al., 2001; Manetsch et al., 2012b; Pemmari et al., 2019; Shah et al., 2014).

Treatment of mice with neutralizing antibody against TNF decreased the expression of *Dusp1* and *Ptprs* and enhanced the resistance of aged mice to pneumococcal challenge—directly implicating TNF as being the causative agent of ADMD and linking it to the immunosuppression observed following steroid therapy. Neutralization of TNF is a highly effective therapeutic intervention in many inflammatory diseases, such as rheumatoid arthritis, ankylosing spondylitis, psoriasis, Crohn's disease, and endotoxin‐induced septic shock (Beutler et al., 1985; Brandt et al., 2000; Chaudhari et al., 2001; Elliott et al., 1993, 1994; Hess et al., 2011; Knight et al., 1993; Lipsky et al., 2000; Lovell et al., 2000; Maini et al., 1998, 1999; Present et al., 1999; Stidham et al., 2014; van Dullemen et al., 1995). Neutralization of TNF has also been recorded to have positive unintended consequences; patients with rheumatoid arthritis treated with anti‐TNF agents had a lower incidence of cardiovascular events and improved insulin resistance (Barnabe et al., 2011; Gonzalez‐Gay et al., 2006; Jacobsson et al., 2005; Kiortsis et al., 2005; van Eijk et al., 2009). Work with experimental animal models has shown that TNF neutralization is an effective therapeutic in heart disease and neurodegenerative diseases like Alzheimer's disease (Csiszar et al., 2007; Moe et al., 2004; Shamim & Laskowski, 2017; Toufektsian et al., 2008; Wolfe & Michaud, 2004). Thus, TNF inhibitors, along with other immune modulators, have possible prophylactic roles that we do not yet fully comprehend. While TNF neutralization can have profound benefits, interfering with the immune system could come with significant risks; serious infections are associated with anti‐TNF therapy, especially the reactivation of latent *Mycobacterium tuberculosis* infections (Cantini et al., 2017; Dixon et al., 2006, 2010; Doran et al., 2002b; Galloway et al., 2011; Gómez‐Reino et al., 2003; Khanna et al., 2004; Listing et al., 2005; Wolfe et al., 2004). Other groups have shown that TNF is essential in fighting off *Spn* infections, as mice from TNF KO backgrounds and mice treated with anti‐TNF could not control the infection successfully (O'Brien et al., 1999; Wellmer et al., 2001). In contrast, we observed that aged mice pretreated with anti‐TNF were less susceptible to infection with decreased bacterial burdens. We purport that the reason for the discrepancy between these reports and our own is that in an excessively inflamed or aged host, anti‐TNF treatment reduces the levels of negative homeostatic suppressors so that an effective cell signaling response to infection can occur at the onset. Whereas during established infection, TNF production is required for the maintenance of a robust immune response to the infection by immune cells.

In summary, chronic exposure to TNF, as during inflamm‐aging, induces the upregulation of established homeostatic suppressors, *Dusp1* and *Ptprs*, of MAPK kinase signaling, which suppresses AM signaling, contributing to the increased susceptibility to infection seen during aging and as a result of corticosteroid therapy. Our results add to the considerable body of evidence implicating TNF as a major determinant of aging, age‐related macrophage dysfunction, and increased susceptibility to infection. Targeting the negative regulators, *Dusp1* and *Ptprs* may prove not to be a viable treatment for aging individuals with pneumococcal infections as they are needed to restrain excessive inflammation, and instead, the focus should be on reducing the root cause of excessive inflammation. Along such lines, exploring new uses of established immune modulators, such as TNF inhibitors, seems more amendable, although with considerable risk. In summary, our findings have enhanced our knowledge of the extent and molecular basis in which TNF is involved in ADMD and susceptibility to infection. They highlight the detrimental consequences of chronic inflammation and suggest that blocking inflammation, in some manner, should be considered as a potential focal point for future interventions of chronic inflammatory and age‐related conditions, such as prophylaxis against infection.

## AUTHOR CONTRIBUTIONS

Conceptualization and Review and Editing: K.L.K., D.M.E.B., and C.J.O. Investigation: K.L.K., E.M., and E.S. Data analysis, K.L.K., E.M., and C.J.O. Supervision: D.M.E.B. and C.J.O.

## CONFLICT OF INTEREST STATEMENT

We have no conflicts of interest to declare.

## Supporting information


Figures S1‐S2.

Table S1.

Table S2.

Table S3.

## Data Availability

The data that support the findings of this study are available from the corresponding author upon reasonable request.
