# Supplementary material for: Chronic TNF exposure induces glucocorticoid‐like immunosuppression in the alveolar macrophages of aged mice that enhances their susceptibility to pneumonia
Source: Aging Cell. 2024 Mar 8;23(6):e14133. doi: 10.1111/acel.14133 (PMC11296116; doi:10.1111/acel.14133)
Supplement: Supplementary file 1 — Figures S1‐S2. [file ACEL-23-e14133-s001.docx]

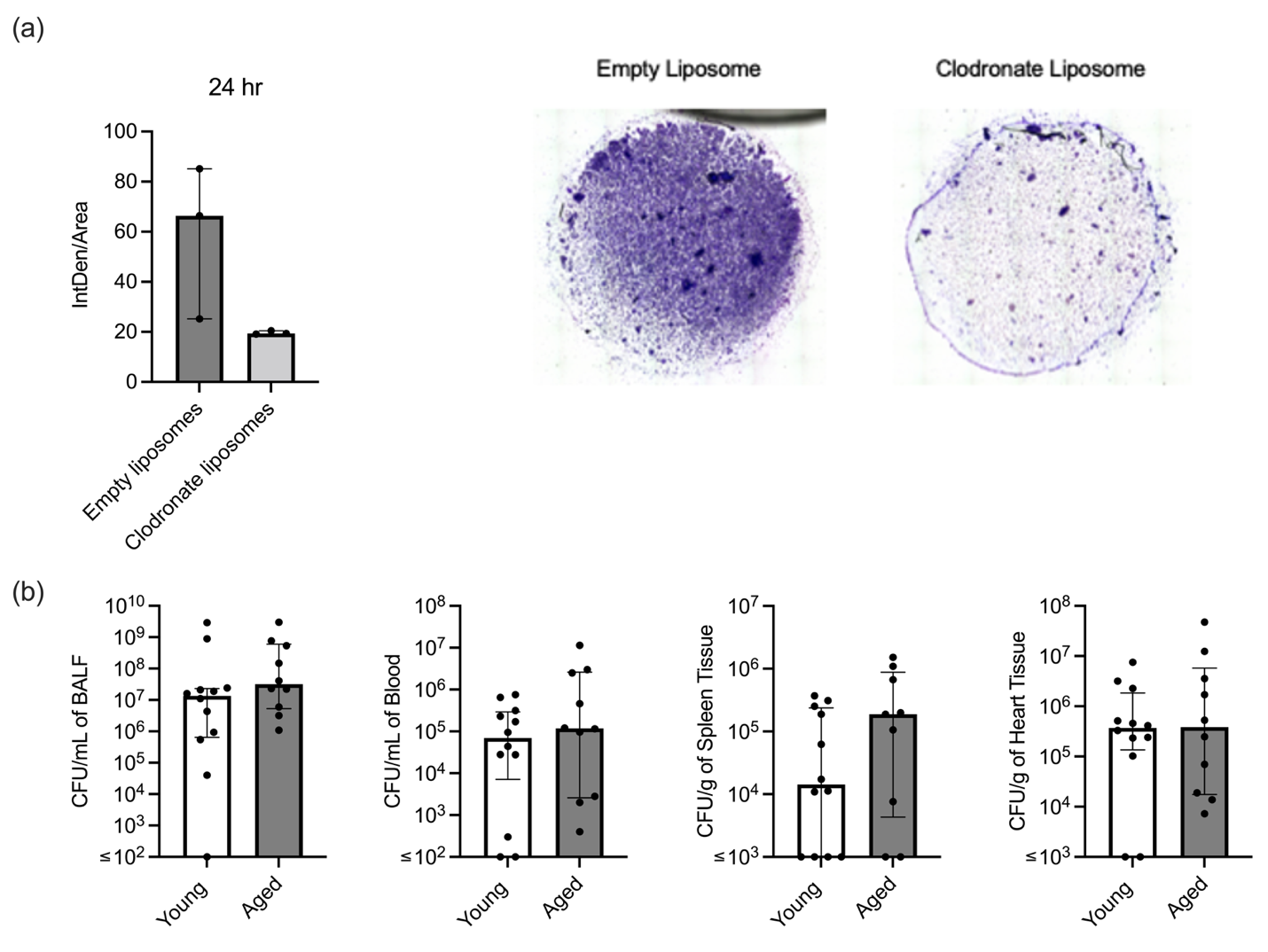


**Supplemental Figure 1. AM depletion and infection of aged mice following adoptive transfer of AM.** To deplete AM, mice were intratracheally administered, by forced inhalation, clodronate liposomes. To validate the efficacy of this treatment, a cohort of mice was euthanized the next day, and BALF was collected with cells spun down onto cytospins slides. The density per area was used as a measure of the number of cells present, which under normal circumstances are principally AM (a). Aged mice were depleted of their own AM, then adoptively transferred with AM from either young or aged mice, and subsequently challenged intratracheally with 10^5^ CFU of *Spn.* Following one day post-infection, the animals were sacrificed, and bacterial burden was enumerated from BALF, blood, spleen, and heart homogenates (b). Statistical significance was calculated using a Mann-Whitney U test (b). Each data point represents an individual mouse. Graphs with ≤ on the x-axis indicate the limit of detection. The data are presented as median with IQR; *p ≤ 0.05.

**Supplemental Figure 2. Neutralizing antibody against TNF reduces TNF levels in the airway.** Mice were treated with a regiment of anti-TNF or anti-HRP control antibodies every other day for two weeks*.* The BALF was collected 24 hours post-challenge. Levels of TNF in BALF were measured by ELISA. Statistical significance was calculated using a Mann-Whitney U test (a). Each data point represents an individual mouse. The data are presented as median with IQR; *p ≤ 0.05.
